# Expression of cytokines in aqueous humor from fungal keratitis patients

**DOI:** 10.1186/s12886-018-0754-x

**Published:** 2018-04-19

**Authors:** Yingnan Zhang, Qingfeng Liang, Yang Liu, Zhiqiang Pan, Christophe Baudouin, Antoine Labbé, Qingxian Lu

**Affiliations:** 1 Beijing Tongren Eye Center, Beijing Tongren Hospital, Capital Medical University, Beijing Ophthalmology & Visual Science Key Lab, Beijing, 100730 China; 2 Beijing Institute of Ophthalmology, Beijing Tongren Eye Center, Beijing Tongren Hospital, Capital Medical University, Beijing Key Laboratory of Ophthalmology and Visual Sciences, Beijing, 100005 China; 3 Quinze-Vingts National Ophthalmology Hospital, Paris and Versailles Saint-Quentin-en-Yvelines University, Versailles, France; 4 INSERM, U968, Paris, F-75012, France; UPMC Univ Paris 06, UMR_S 968, Institut de la Vision, Paris F-75012, France; CNRS, UMR_7210, Paris F-75012, France, Paris, France; 5 Department of Ophthalmology and Visual Sciences, University of Louisville, 301 E. Muhammad Ali Blvd, Louisville, KY 40202 USA

**Keywords:** cytokines, aqueous humor, fungal keratitis, inflammation

## Abstract

**Background:**

Although a series of reports on corneal fungal infection have been published, studies on pathogenic mechanisms and inflammation-associated cytokines remain limited. In this study, aqueous humor samples from fungal keratitis patients were collected to examine cytokine patterns and cellular profile for the pathogenesis of fungal keratitis.

**Methods:**

The aqueous humor samples were collected from ten patients with advanced stage fungal keratitis. Eight aqueous humor samples from patients with keratoconus or corneal dystrophy were taken as control. Approximately 100 μl to 300 μl of aqueous humor in each case were obtained for examination. The aqueous humor samples were centrifuged and the cells were stained and examined under optical microscope. Bacterial and fungal cultures were performed on the aqueous humor and corneal buttons of all patients. Cytokines related to inflammation including IL-1β, IL-6, IL-8, IL-10, TNF-α, and IFN-γ were examined using multiplex bead-based Luminex liquid protein array systems.

**Results:**

Fungus infection was confirmed in these ten patients by smear stains and/or fungal cultures. Bacterial and fungal cultures revealed negative results in all aqueous humor specimens. Polymorphonuclear leukocytes were the predominant infiltrating cells in the aqueous humor of fungal keratitis. At the advanced stages of fungal keratitis, the levels of IL-1β, IL-6, IL-8, and IFN-γ in the aqueous humor were significantly increased when compared with control (p<0.01). The levels of IL-10 and TNF-α also showed an ascending trend but with no statistical significance.

**Conclusions:**

High concentration of IL-1β, IL-6, IL-8, and IFN-γ in the aqueous humor was associated with fungal keratitis.

## Background

Corneal infection is one of the major eye diseases affecting public health worldwide [1], especially in developing countries [2]. Its increasing incidence and the difficulty associated with therapy result in severe vision problems or blindness [3]. The incidences of fungal keratitis are higher in the harvest seasons. Males and the middle-aged (41–50 years old) population are more likely to be affected by fungal keratitis. The cornea is particularly affected by fungal infections due to its susceptibility to injury and non-vascularization, resulting in lower resistance. The major pathogenic fungi are *Fusarium* species (73.3%), followed by *Aspergillus* species (12.1%) [4].

Although a series of reports on corneal fungal infection have been published including epidemiology, diagnostics, pathogenic risk factors, and treatment methods, studies on pathogenic mechanisms and inflammation-associated cytokines remain relatively limited [5, 6]. Research on the pathogenesis of fungal keratitis has been performed on animal models [7, 8] and on the tear samples of the patients with fungal keratitis [9]. However, tears are highly susceptible to environment and ophthalmic medication due to their surface location.

Clinically, we found that some fungal keratitis patients experienced hypopyon and fibrin exudation in the anterior chamber, but their corneal endothelia were clear. After deep anterior lamellar keratoplasty (DALK), the hypopyon disappeared without recurrence of infection. It has been suggested that cytokines in aqueous humor play an important role in the pathogenesis of fungal keratitis.

In the present study, aqueous humor samples from fungal keratitis patients were collected to monitor the intraocular inflammatory response. Cytokine patterns and cellular profile were analyzed for pathogenesis of fungal keratitis.

## Methods

### Patients

This study was approved by the Medical Ethical Committee of Beijing Tongren Hospital. Before surgery, informed consents were obtained from the patients and parents/legal guardians (if the patient ≤18 years old) of all the participants after explanation of the nature and possible consequences of the study.

Ten patients of clinically diagnosed fungal keratitis and eight patients with keratoconus or corneal dystrophy who had undergone penetrating keratoplasty in Beijing Tongren Hospital from June to November of 2014 were recruited for this study.

The cases were diagnosed as fungal keratitis when fungal culture and/or scraped specimen staining were positive. Patients who did not respond to initial therapy with topical and systemic antifungal drugs were recruited. A detailed clinical and demographic history was taken and a thorough slit-lamp biomicroscopic examination was performed for all the patients. The size, depth, and margins of the ulcer were noted, along with the presence of satellite lesions and hypopyon height. The presence of an epithelial defect and pigmentation on the surface was also recorded.

### Samples Collection

Before surgery, the corneal culture specimens and scrapings were taken from the base and edge of the ulcers aseptically with sterile cotton-tipped swabs and placed in transport medium. The scraped specimens were sent for inoculation in Sabouraud’s dextrose agar medium for fungal culture. All the corneal scrapings were also sent for routine Gram’s stain and bacterial culture in nutrient broth.

The patients received penetrating keratoplasty with general anesthesia. The donor corneas were obtained from Beijing Tongren Eye Bank. Paracentesis lancet was used to penetrate the cornea in an avascular peripheral area over a length of 1 mm. Thereafter, approximately 100 μl to 300 μl of aqueous humor was taken out of the anterior chamber without contact with intraocular structures. Each sample of aqueous humor was centrifuged for 10 min at 2000  revolutions per minute (rpm) to separate cells from fluid. Reserved 50 μl supernatants were transferred into sterile microfuge tube and stored at − 80 °C until assaying for cytokine, and the remaining supernatant was used for bacterial and fungal culture. The cell pellet was resuspended in 200 μl of phosphate buffered saline and deposited onto glass slides. After air-drying, the cells were stained with Giemsa and examined by optical microscopy. The cells were counted and morphologically classified into polymorphonuclear leukocytes (PMN), lymphocytes, and monocytes.

In all cases, the corneal buttons of patients, which were obtained at the time of penetrating keratoplasty, were sent for microbial investigations.

### Microbiological Culture

The aqueous humor and cornea from all the patients were inoculated on culture medium at 28 °C with a humidity of 40% for 8–10 days. The culture medium contained Sabouraud’s agar and potato glucose agar. The fungi were identified according to the characteristics of growing colonies, hyphae, and spore. In addition, the specimens of aqueous humor and cornea were inoculated on broth medium at 37 °C with a humidity of 40% for 10–14 days for bacteria identification.

### Cytokines Measurement by Liquid Protein Array System

The levels of cytokines including interleukin (IL)-1β, IL-6, IL-8, IL-10, interferon-γ (IFN-γ), and tumor necrosis factor-α (TNF-α) in the aqueous humor were measured using Luminex100™ liquid protein array systems (MiraiBio, CA, US).

### Statistical Analysis

Statistical analyses were performed by SPSS software (version 11.5, SPSS Inc., Chicago, Illinois, USA). The levels of each cytokine were compared by means of the data and reported as medians with minimum and maximum levels obtained for each group. The results are presented as the geometric mean concentration and the range of detectable samples (Table 2). The cytokine levels were compared between two groups with nonparametric two-sample median test. A *p*-value of < 0.05 was considered to be significant.

## Results

### Patient Cohort

The average age of patients with fungal keratitis was 49.30 ± 17.02 years (range from 15 to 72 years old). Three patients (30%) were female and seven patients (70%) were male. Five eyes (50%) had a history of wooden foreign body or plant injury. In addition to antifungal therapy, four eyes (40%) had been treated with topical corticosteroid or corticosteroid-antibiotic combination therapy and three eyes (30%) had been treated with topical antibiotics only.

The control patients with keratoconus (87.5%) or corneal dystrophy (12.5%) received penetrating keratoplasty for visual restoration. Their average age was 24.88 ± 12.12 years (range from 11 to 50 years old). Two patients (25%) were female and six patients (75%) were male. (Table 1).

**Table 1:** Demographic, clinical and microbiological aspects in fungal keratitis patients and controls

No.	Gender	Age range (years old)	Diagnosis	Duration (days)	Pathogeny	Smear (fungus)	Aqueous humor culture (fungus / bacteria)		Corneal culture (fungus / bacteria)		Ulcer (mm × mm)	Hypopyon (depth, mm)
1	M	11–20	FK	7	None	+	–	–	+	–	6 × 5	2.0
2	F	51–60	FK	7	Foreign body	+	–	–	+	–	8 × 7	2.0
3	F	41–50	FK	7	None	–	–	–	+	–	5 × 4	1.0
4	F	41–50	FK	18	None	+	–	–	+	–	7 × 6	4.0
5	M	61–70	FK	30	None	+	–	–	+	–	5 × 4	1.0
6	M	41–50	FK	10	Plant Injury	+	–	–	+	–	3 × 3	0.5
7	M	41–50	FK	10	Foreign body	–	–	–	+	–	8 × 7	2.0
8	M	71–80	FK	30	Plant Injury	–	–	–	+	–	6 × 5	5.0
9	M	71–80	FK	12	None	–	–	–	+	–	5 × 4	3.0
10	M	41–50	FK	30	Plant Injury	–	–	–	+	–	4 × 3	2.0
11	F	11–20	KC				–	–	–	–		
12	F	41–50	CD				–	–	–	–		
13	M	11–20	KC				–	–	–	–		
14	M	11–20	KC				–	–	–	–		
15	M	21–30	KC				–	–	–	–		
16	M	11–20	KC				–	–	–	–		
17	M	31–40	KC				–	–	–	–		
18	M	21–30	KC				–	–	–	–		

Note: *M*: male; *F*: female; *FK*: fungal keratitis; *KC*: keratoconus; *CD*: corneal dystrophy; +, positive; −, negative

### Characterization of Fungal Keratitis

Therapeutic keratoplasty had been taken since the patients did not respond to the medication treatment, with a tendency to corneal melting and perforation. The time course of disease development was ranged from 7 to 30 days, with an average of 15.89 ± 10.72 days. Epithelial defect and pigmentation on the surface, characterized as dry and pigmented ulcers with irregular and feathery margins, satellite lesions, fibrinoid aqueous reaction, and hypopyon formation, were present on the infected eyes of fungal keratitis patients. The features of ulcers are highlighted in Table 1.

### Cytopathologic Examination

The PMN cell populations were the predominant infiltrated cells types in the aqueous humor samples collected from the infected eyes of fungal keratitis patients. A low percentage of lymphocytes and monocytes were also observed. The percentage of these infiltrating cells is depicted in Table 2. The difference in the pattern of infiltrating cells between fungal keratitis and the control was statistically significant (*p* < 0.001 for each population).

**Table 2:** Percentage of various infiltrating cells in aqueous humor from fungal keratitis patients and controls

	% Polymorphonuclear			% Lymphocytes			% Monocytes		
	Median	Minimum	Maximum	Median	Minimum	Maximum	Median	Minimum	Maximum
FK	89.5	85.7	92.0	8.1	6.5	13.2	3.9	0.5	6.8
Control	0	0	0	0	0	0	0	0	0
*p*	< 0.001			< 0.001			< 0.001		

Note: *FK*: fungal keratitis

### Microbial Investigation

The positive fungal infection rate in the keratitis patients was 50% by smear staining and 100% by corneal culture. Among ten positive cases of corneal fungal culture, strain *Fusarium spp*. was evident in six cases (60%), strain *Aspergillus spp*. was present in two cases (20%), and strain *Apospory spp*. was identified in the other two cases (20%). However, it was noteworthy that the aqueous humor cultures from both fungal keratitis and control groups showed likewise negative presentation of either fungus or bacteria.

### Cytokine Profiles

The protein levels of cytokines IL-1β, IL-6, IL-8, IL-10, IFN-γ, and TNF-α were measured by Liquid Protein Array System. The concentration of six cytokines in the control group was used as a basal level for comparison. The cytokine levels showed remarkable difference between fungal keratitis and control group. In the aqueous humor samples of fungal keratitis group, the levels of IL-1β, IL-6, IL-8, and IFN-γ were found to be significantly increased, as compared with the control group (*P* = 0.012 for IL-1β, *P* < 0.001 for IL-6, P < 0.001 for IL-8, and *P* = 0.001 for IFN-γ). Although IL-10 and TNF-α levels were also elevated, they showed no statistically significant difference (Table 3).

**Table 3:** Cytokine levels in aqueous humor from fungal keratitis patients and controls

Cytokine levels (pg/ml)	Fungal keratitis (*n* = 10)		Control (*n* = 8)		
	Mean (±SD)	Median	Mean (±SD)	Median	*p*
IL-1β	172.89 ± 45.83	121.41	7.50 ± 3.22	5.98	0.012
IL-6	6179.71 ± 1015.726	6712.84	6.22 ± 7.55	3.12	0.000
IL-8	13,003.82 ± 1803.97	13,755.86	9.61 ± 9.24	6.17	0.000
IL-10	25.32 ± 18.99	26.13	8.32 ± 0.25	8.23	0.230
TNF-α	22.82 ± 56.69	1.92	1.52 ± 2.10	0.00	0.237
IFN-γ	28.70 ± 18.93	7.91	2.32 ± 1.18	3.17	0.001

## Discussion

The aqueous humor cultures from ten fungal keratitis patients showed negative in fungal and bacterial infection, it further confirmed that a sterile reaction occurred in the aqueous humor of some fungal keratitis patients despite hypopyon. The major infiltrating cells in the aqueous humor were PMN leukocytes. Based on the animal experiments and clinical corneal histology, the PMN leukocytes in the aqueous humor are considered the major cellular basis of fungal keratitis [7, 10]. Our present results confirm this expectation. It is likely that these infiltrating cells are involved in the clearance of pathogens [11]. In contrast, no inflammatory cells were found in the control.

In the present study, we measured and analyzed the intraocular cytokine profiles in relation to fungal keratitis. During the middle-advanced stage of fungal infection, the IL-1β, IL-6, IL-8, and IFN-γ levels in the aqueous humor were significantly increased compared to non-keratitis controls.

Cytokine levels in aqueous humor have been reported as indicators of local ocular immunological processes. Increased IL-1β and IL-6 are the specific inflammatory signals for keratohelcosis and keratitis [12]. IL-1β, IL-6, and IL-8 are significantly increased in the tears of bacterial keratitis patients, along with accumulating dendritic cells [13]. In studies of animal models on the herpes simplex virus-infected keratitis, cytokines such as IL-1β, IL-6, IL-8, IL-10, IL-12, and IFN-γ were demonstrated to play a predominant role in disease development [14, 15]. In corneal epithelial cells infected with *Pseudomonas*, IL-1β functioned as the major inflammatory mediator regulating IL-6 and IL-8 expression [16]. IL-6, IL-10, and IFN-γ were elevated in acute uveitis aqueous humor. IL-10 was increased in infective uveitis compared with non-infective uveitis [17]. Cytokines IL-4, IFN-γ, and TNF-α were increased in aqueous humor in Behçet’s uveitis [18, 19]. IL-6, IL-10, and IFN-γ were involved in rejection after corneal transplantation [20]. All these reports indicate that the expression of cytokines is closely related to the ocular inflammatory and immunologic reactions.

IL-1β is a proinflammatory cytokine and an important inflammation mediator. In the inflammatory reaction, it can induce synthesis of other cytokines, activation of T lymphocyte, and migration of monocyte, macrophage, and Langerhans’ cells [21, 22]. In our study, a high level of IL-1β was observed in the aqueous humor samples collected from fungal keratitis patients. It is quite possible that the elevated level of IL-1β caused severe leukocyte infiltration and blood-aqueous barrier damage. IL-6 is a potential mediator of intraocular inflammation, and several evidence indicate that it plays an important multifunctional role in corneal infection and inflammation [23]. Moreover, it can activate the production of antibody and fibrous proteins, induce the production of proteins in acute inflammation, and serve as an activator of macrophage and chemotactic factors for T lymphocytes [24]. IL-6 can also be induced by other cytokines such as IL-1β, TNF-α, and IFN-γ and be released by retinal pigment epithelial cells, corneal endothelial cells, macrophage, iris, and ciliary body epithelial cells [25]. IL-8 selectively activates PMN leukocytes and T cells and imposes a chemotactic effect on neutrophilic granulocytes, which are induced to infiltrate into the inflammatory sites, thus increasing vascular permeability and activating cytokines (such as IL-1β and TNF-α) to be released. As a consequence, such inflammatory responses further lead to a continuous increase in IL-8 level and accumulation of leukocytes, which exacerbates inflammation [26, 27]. Therefore, the increased IL-8, as observed in the aqueous humor of the fungal keratitis patients, could be a primary cause for the increased PMN infiltration and the high expression level of IL-1β. IFN-γ is produced by activated lymphocytes. It can inhibit cell proliferation and prevent other cytokines from being recruited to sites of inflammation. IFN-γ is a primary cytokine involved in delayed type hypersensitivity [28]. IFN-γ in the aqueous humor of the fungal keratitis patients is secreted by the increased infiltrating lymphocytes, which, to some extent, might inhibit the inflammation severity.

IL-10 and TNF-α levels in the aqueous humor of fungal keratitis patients were higher on average than those of the control, but with no statistical differences. IL-10 is an anti-inflammatory cytokine. IL-10 could represent an inhibitory factor in the T helper 1 cells response. Indeed, IL-10 takes protective effects during inflammation as an inflammatory cytokine-inhibiting factor [28, 29]. TNF-α is an important factor in connecting specific immunity and inflammatory reaction. It is mainly produced by activated monocytes and can induce monocytes to synthesize IL-1β, IL-6, and IL-8 [30].

Our subjects involved only a small number of patients, followed over a limited period of time, and the cytokines analyzed were restricted to the inflammation related cytokines. The cytokine and inflammatory cells infiltrate profiles in the aqueous humor in keratitis caused by other pathogenic microorganisms have not been reported previously. In the present study, patients with keratoconus and corneal dystrophy were selected as the negative control. Though strictly they may serve as a control for non-inflammatory ocular pathologies. Further investigations are needed to report on the roles of cytokines and other cell signal transduction factors in each stage of inflammation in fungal keratitis. Due to the diverse roles of cytokine in different signal transduction pathways and additional biological effects caused by interactions between cytokines and its productions, studies of the cytokine network are more meaningful than that of single cytokines.

Since cytokines in aqueous humor play important roles in the pathogenesis of fungal keratitis, the use of intervention strategies in related cytokines (such as blocking cytokines binding to receptors, or competitive binding to receptors using inactivated cytokine analogs and blocking their functions after binding) to achieve therapeutic goals is worthy of further investigation.

## Conclusions

The present studies demonstrate that a high concentration of IL-1β, IL-6, IL-8, and IFN-γ in the aqueous humor is associated with fungal keratitis, and the infiltrating PMN leukocytes are involved in this inflammatory response. Studying the cytokine profile in the aqueous humor of fungal keratitis patients is beneficial for elucidation of pathological changes and inflammatory responses of fungal keratitis. It is anticipated that further studies in this direction will lead to innovation and development of more effective therapeutic strategies for fungal keratitis.

## Abbreviations

IFN-γ: Interferon-γ
DALK: Deep anterior lamellar keratoplasty
IL: Interleukin
PMN: Polymorphonuclear leukocytes
rpm: Revolutions per minute
TNF-α: Tumor necrosis factor-α
